# Expressional and prognostic value of HPCAL1 in cholangiocarcinoma via integrated bioinformatics analyses and experiments

**DOI:** 10.1002/cam4.4897

**Published:** 2022-05-29

**Authors:** Mingjian Ma, Guangyan Zeng, Jinhui Li, Jiahua Liang, Li Huang, Jiancong Chen, Jiaming Lai

**Affiliations:** ^1^ Department of Pancreato‐Biliary Surgery First Affiliated Hospital, Sun Yat‐Sen University Guangzhou PR China; ^2^ Department of Gastrointestinal Surgery Eighth Affiliated Hospital, Sun Yat‐sen University Shenzhen PR China; ^3^ Department of Pharmacology and Experimental Therapeutics Boston University School of Medicine Boston Massachusetts USA

**Keywords:** cholangiocarcinoma, HPCAL1, integrated bioinformatics analyses, prognostic biomarker

## Abstract

**Background:**

Hippocalcin‐like 1 (HPCAL1) is involved in the development of several cancer types. However, our understanding of the HPCAL1 activity in cholangiocarcinoma (CCA) remains limited.

**Methods:**

Two microarray datasets were used to screen for differentially expressed genes (DEGs) involved in the development of CCA. The Cancer Genome Atlas (TCGA)/Gene Expression Omnibus (GEO) database was integrated to determine the prognostic significance of DEGs in CCA. The association between clinical characteristics and HPCAL1 expression levels was initially explored to assess the clinical profile of CCA. The prognostic value of HPCAL1 overexpression in the validation cohort was analyzed, followed by Gene Ontology (GO) term analysis and Kyoto Encyclopedia of Genes and Genomes (KEGG) pathway analysis of HPCAL1.

**Results:**

Three upregulated genes and 10 downregulated genes were detected from two microarray‐based screenings. High expression of HPCAL1 as a poor prognostic factor of CCA was validated using TCGA/GEO integrated database and our database. Univariate and multivariate analyses along with Kaplan–Meier survival analysis showed that high HPCAL1 expression was an independent factor affecting the overall survival and relapse‐free survival in patients with CCA. The high expression of HPCAL1 was significantly associated with cancer antigen 125 (CA‐125) levels, number of tumors, lymph node invasion, and TNM stage. Analysis of the enriched GO terms and KEGG pathways revealed that the high expression of HPCAL1 was involved in the critical biological processes and molecular pathways, including modulation by a host of symbiont processes, the clathrin coat, actinin binding, and Rap1 signaling pathways.

**Conclusion:**

HPCAL1 was enriched in CCA in our study and has the potential to be applied in the identification of patients with CCA with an unfavorable prognosis.

## Graphical abstract

(A) IHC staining for HPCAL1 (B) Multivariate Cox analysis of clinicopathological variables and OS/RFS (C) Overall survival and relapse‐free survival in CCA patients with different levels of HPCAL1.

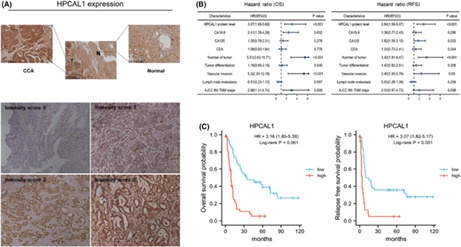

## 1 INTRODUCTION

Cholangiocarcinoma (CCA) is the second most common primary hepatocarcinoma^1^ and is anatomically classified into intrahepatic cholangiocarcinoma (iCCA) and extrahepatic cholangiocarcinoma (eCCA).^2^ Usually, patients with CCA do not present remarkable symptoms in the early stages,^1^ and the pathogenesis remains unclear.^3^ The incidence of CCA has continued to increase in Western countries for several years^4^: the incidence of CCA has increased from 0.49 per 100,000 in 1995 to 1.49 per 100,000 in 2014, with an average annual increase rate of 5.49%,^2^ while it was over six per 100,000 in South Korea, China, and Thailand until 2020.^5^ Additionally, more than 66% of patients are ineligible for surgery at diagnosis, while more than 60% experience relapse after surgery.^2^ Therefore, more reliable and specific prognostic biomarkers are urgently required for patients diagnosed with CCA.

Due to the rapid advancement of whole‐genome sequencing technology and free access to high‐throughput oncology databases, bioinformatics data mining or validation has been widely applied to various fields of oncology.^6^, ^7^ Large‐scale genomics projects, such as The Cancer Genome Atlas (TCGA, http://cancergenome.nih.gov/) and Gene Expression Omnibus (GEO, http://www.ncbi.nlm.nih.gov/geo/), have greatly facilitated data mining discoveries and biomedical research through the generation, management, and public release of multiple omics datasets collected from thousands of samples.^8^, ^9^ The convenient access to big data (TCGA and GEO databases) allows large‐scale gene expression profiling to explore the potential correlations between genes and the prognosis of CCA.

Hippocalcin‐like 1 (HPCAL1), also known as VILIP3, has been identified in the central nervous system (CNS) as multiple calcium‐sensing proteins.^10^ Due to its downregulation in tissues and cells, HPCAL1 was recognized as a new suppressor gene for hepatocellular carcinoma (HCC) and was found to aggravate the clinical outcomes in HCC patients.^11^ A previous study found that neurite outgrowth can be eliminated by knocking down HPCAL1 expression in neuroblastoma cells.^12^ Currently, the mechanisms underlying the expression of HPCAL1 in CCA pathogenesis are not fully understood.

In this study, two microarray datasets (GSE34166 and GSE22633) from the GEO database were used to screen for differentially expressed genes (DEGs). By employing two independent patient cohorts (integrated TCGA‐CHOL/GEO‐GSE107943 database and our single‐center CCA cohort) to verify the expression and prognosis of DEGs, we identified a novel proto‐oncogene, HPCAL1, involved in CCA. Moreover, the relationship between HPCAL1 expression and clinicopathological characteristics was explored further. Gene Ontology (GO) and Kyoto Encyclopedia of Gene and Genome (KEGG) pathway analyses were performed to analyze the molecular function (MF), cellular component (CC), biological process (BP), and KEGG pathways of HPCAL1. Therefore, bioinformatics methodology was adopted to explore the potential mechanisms and clinical values of the biomarker genes identified in the prognosis and carcinogenesis pathway of CCA and provide more evidence for the development of therapeutic targets and clinical applications.

## 2 MATERIALS AND METHODS

### 2.1 Integrational analysis of microarray datasets

Public gene expression data and full clinical annotation were searched in the GEO and TCGA databases. The RNA expression data for DEGs were extracted from the GEO datasets (https://www.ncbi.nlm.nih.gov/geo) (GSE34166, GSE22633). The RNA sequencing data (FPKM value) of gene expression were downloaded from TCGA‐CHOL database and GEO database under the accession number GSE107943. Then, these two datasets (GSE107943 and TCGA‐CHOL) were integrated and normalized using the R 3.6 software (sva and limma packages). Patients with data on histologically diagnosed CCA, who did not receive neoadjuvant chemotherapy or radiotherapy, and with complete survival information were eligible for the study. A total of 63 patients with CCA were recruited for the prognostic analysis of the overall survival and relapse‐free survival.

### 2.2 Patients and clinical samples

Patients with pathologically confirmed CCA (*n* = 84) from the First Affiliated Hospital of Sun Yat‐sen University between April 2007 and August 2012 were enrolled in this clinicopathological correlation analysis. Patients (i) who were pathologically diagnosed with CCA, (ii) who underwent radical surgical resection, and (iii) with complete clinicopathological and follow‐up data were included in the study. Those (i) who underwent preoperative chemotherapy or radiotherapy, (ii) with concomitant malignancies of other organs, and (iii) who underwent pathologically proven non‐curative resection with positive margins were excluded. The study was conducted in accordance with the Declaration of Helsinki and approved by the Ethics Committee of the First Affiliated Hospital of Sun Yat‐sen University. Written informed consent was obtained from all patients.

### 2.3 Western blot

CCA samples were ground and lysed using RIPA buffer (Beyotime) along with protease inhibitor cocktail (CoWin Biosciences) for 30 min. A BCA Protein Assay Kit (Thermo Fisher Scientific) was used to measure the protein concentration. Approximately 20 μg of protein was loaded in 12.5% sodium dodecyl sulfate‐polyacrylamide gel electrophoresis and then transferred to a 0.45‐μm polyvinylidene difluoride membrane. The transfer membranes were blocked in 1× protein‐free rapid blocking buffer (Epizyme Biotech) for 10–15 mins. After shaking with primary detection antibody (HPCAL1, 22 kDa, Proteintech) gently overnight at 4°C, the membranes were incubated with the corresponding secondary detection antibodies for 1–2 h at room temperature. Chemiluminescent HRP substrate (Millipore) was used to detect the binding of antibody to antigen. Finally, β‐tubulin (Abcam, 55 kDa) was used as an internal reference. The protein bands from western blotting were subjected to ImageJ software analysis.

### 2.4 Real‐time qPCR

TRIzol reagent (Invitrogen) was used to extract total RNA from the CCA samples. cDNA synthesis was performed using the SureScript First‐Strand cDNA Synthesis Kit (GeneCopoeia) according to the manufacturer's protocol. Real‐time quantitative polymerase chain reaction (RT‐qPCR) was performed using the BlazeTaq™ One‐Step SYBR® Green RT‐qPCR Kit (GeneCopoeia) on a ViiA 7 RT‐PCR System (Applied Biosystems). GAPDH was used for normalization.

### 2.5 Immunohistochemistry

Immunohistochemistry (IHC) analysis was performed on 84 CCA tissues to detect the presence of HPCAL1 protein. Paraffin‐embedded tissue sections were then prepared and baked. The slides were deparaffinized in xylene and rehydrated in graded alcohol. Hydrogen peroxide (3%) was used for blocking followed by antigen retrieval. The tissues were then incubated with VILIP3 polyclonal antibody (dilution 1:200; also known as HPCAL1, Proteintech) at 4°C overnight. The staining intensity (negative, 0; mild, 1; moderate, 2; and strong, 3) and percentage area of positive cells (≤25, 1; >25 and ≤50%, 2; >50 and ≤75%, 3; and >75%, 4) were quantified, respectively.^13^ Staining was independently evaluated by two experienced, blinded pathologists at the First Affiliated Hospital, Sun Yat‐sen University, Guangzhou, China. The scores of the two groups were summed to obtain the final IHC scores.

### 2.6 Go and KEGG pathway analysis

GO enrichment, consisting of cellular components, molecular functions, and biological process, defines the unique biological characteristics of certain genes from different aspects. The KEGG is an enrichment analysis used to investigate the biological pathways involved in certain genes. Here, the R 3.6 software with “clusterProfiler” package, which was applied to perform GO and KEGG analysis, was used. Moreover, “ggplot2” package was used for the outcome visualization.

### 2.7 Statistical analysis

Statistical analyses were performed using SPSS 22.0, GraphPad Prism 8.0, and R version 3.6. Student's *t*‐test and analysis of variance were used to compare continuous variables between the two groups or among multiple groups. Categorical variables were compared using the chi‐square test, continuity correction test, or Fisher's exact test. Survival curves were obtained using the Kaplan–Meier method, while statistical significance was assessed using the log‐rank test. A *p* value of <0.05 was considered significant.

## 3 RESULTS

### 3.1 Microarray data and identification of DEGs in CCA

**FIGURE 1:**
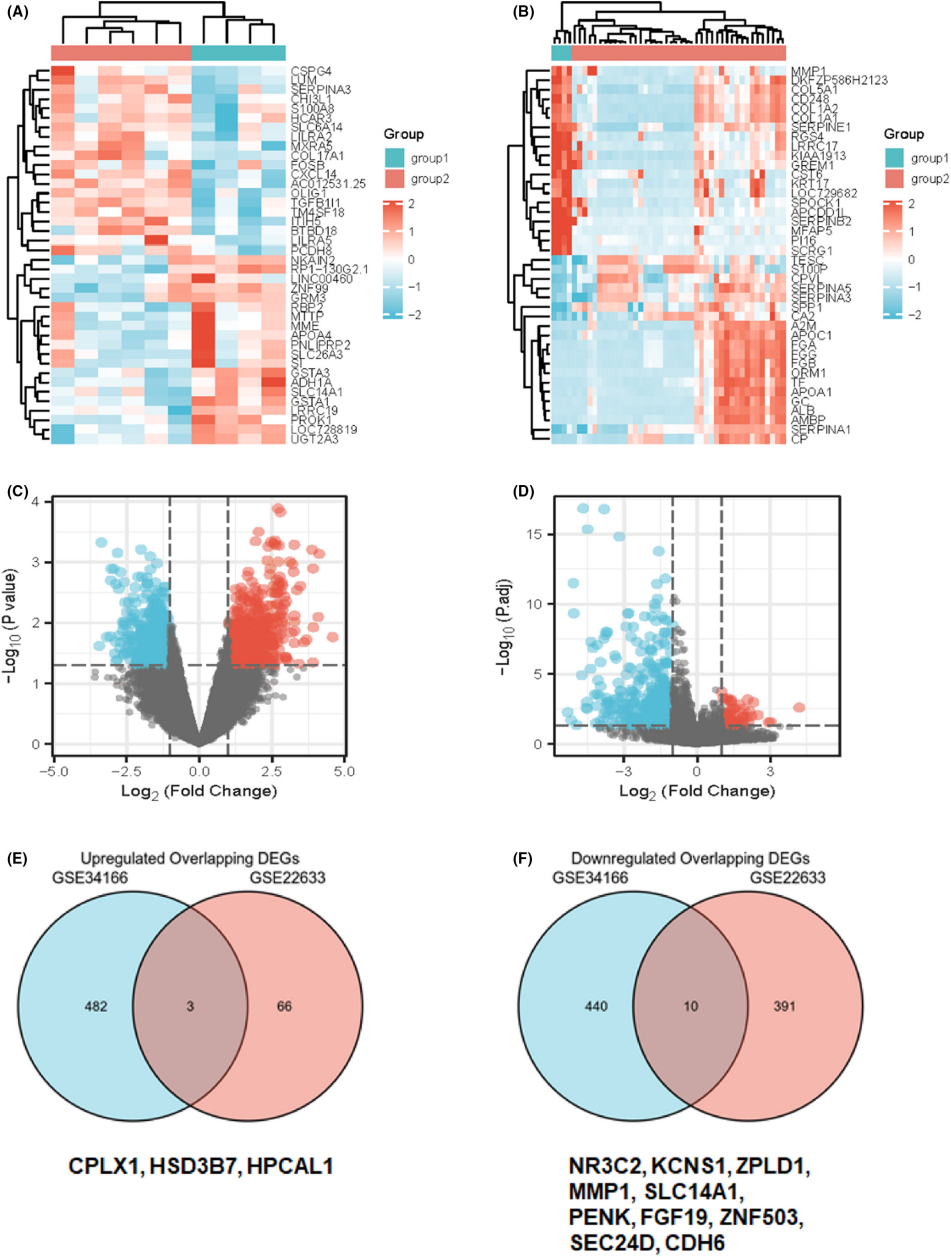
Screening DEGs in CCA. (A, B) Heatmap of top 20 DEGs in GSE34166 and GSE22633. (C, D) Volcano plot of DEGs in GSE34166 and GSE22633. (E, F) Venn diagram of upregulated and downregulated overlapping DEGs.

To screen for DEGs in CCA, two microarrays were selected from the tissue and cell aspects for data mining. In the current study, six CCA cancer tissues and four normal bile duct controls from the GSE34166 dataset were included. Finally, 485 upregulated and 450 downregulated genes were identified (|log FC| ≥ 1, *p* < 0.05). Similarly, 42 CCA cells and four normal biliary epithelial cells from the GSE22633 dataset were used, of which 69 upregulated genes and 401 downregulated genes were screened. The top 20 upregulated and downregulated genes are shown in heat maps (Figure 1A,B). A volcano plot was used to visualize the overall distribution of the DEGs (Figure 1C,D). Next, the interaction of DEGs from the two microarrays was obtained using the Venn diagram (Figure 1E,F), including three upregulated genes (CPLX1, HSD3B7, and HPCAL1) and 10 downregulated genes (NR3C2, KCNS1, ZPLD1, MMP1, SLC14A1, PENK, FGF19, ZNF503, SEC24D, and CDH6).

### 3.2 Prognostic significance of DEGs in CCA identified from TCGA/GEO database

**FIGURE 2:**
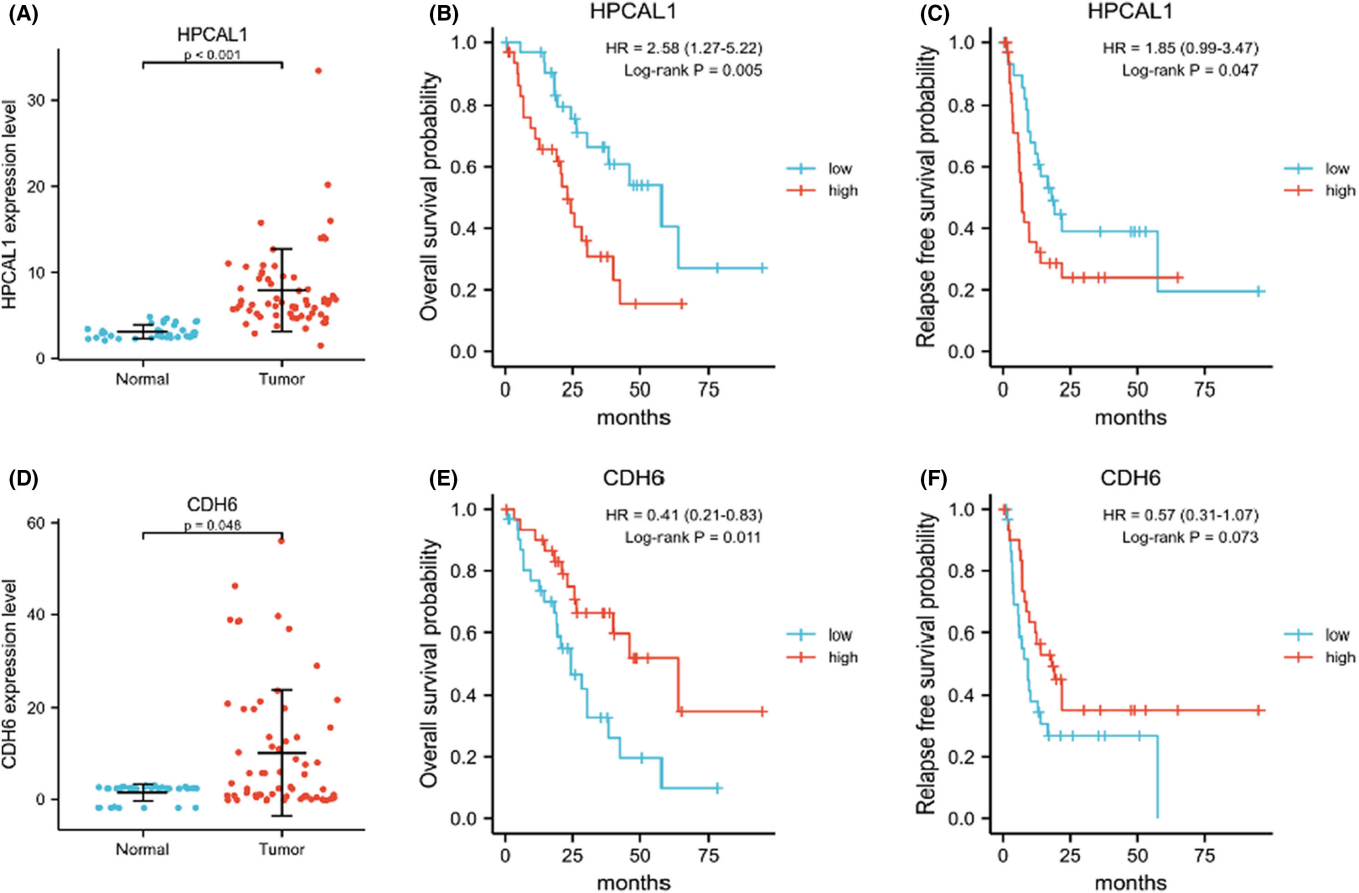
Differential expression and survival analysis of the screening genes. (A–C) Expression, Overall survival probability, and Relapse‐free survival probability of HPCAL1. (D–F) Expression, Overall survival probability, and Relapse‐free survival probability of CDH6.

For further investigation, data from TCGA‐CHOL and GEO‐GSE107943 cohorts were collected and integrated. All DEGs screened above were counted. Figure S1 shows a list of the identified DEGs. HPCAL1 and CDH6 were the significantly upregulated and downregulated genes in the datasets (Figure 2A,D). HPCAL1 presented a remarkably poor overall survival (OS) and relapse‐free survival (RFS) probability along with a significant differential expression in CCA (Figure 2B,C), whereas the differential expression trend and RFS probability of CDH6 in CCA were disappointing (Figure 2E,F). Moreover, the association between other gene regulation changes and the risk of OS and RFS is illustrated in Figures S2 and S3.

### 3.3 Relationship between clinicopathological characteristics and HPCAL1 expression

**FIGURE 3:**
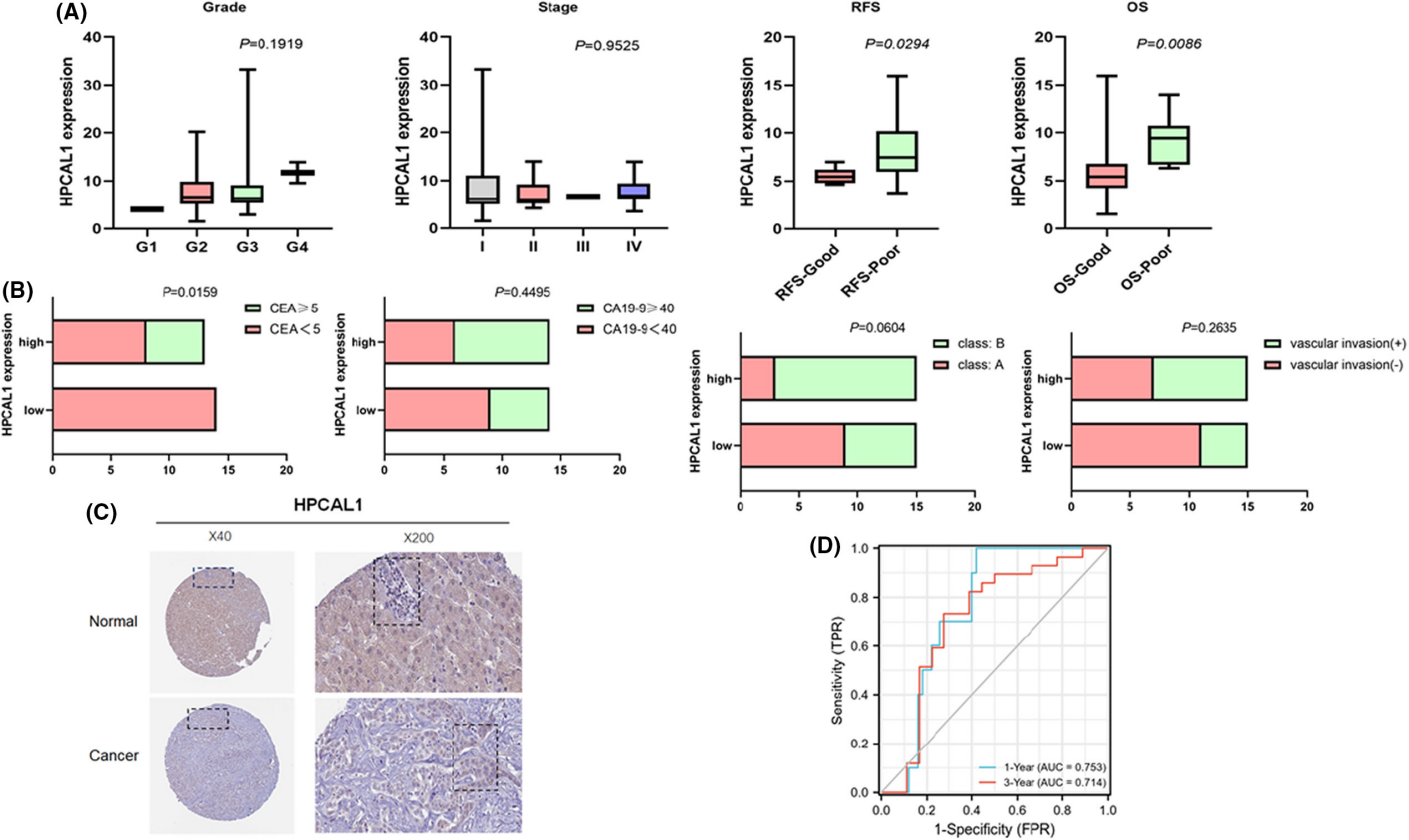
The relationship between HPCAL1 and the clinicopathological characteristics. (A) The clinical significance of HPCAL1 toward grade, stage, and prognosis. (B) The correlation of HPCAL1 and CEA, CA19‐9, Child‐Pugh class and vascular invasion. (C) Cholangiocarcinoma expression of HPCAL1 protein by IHC in HPA database. (D) ROC curves of HPCAL1 of 1 year and 3 year.

The clinical significance of HPCAL1 expression was evaluated using the integrated TCGA‐CHOL/GEO‐GSE107943 database. As shown in Figure 3A, high HPCAL1 expression was correlated with poor OS and RFS (criteria: good ≥3 years, living/relapse‐free, and poor ≤1 year, death/relapse prognosis) but was not significantly correlated with tumor histologic grade and clinical stage. Based on the GSE107943 database, HPCAL1 expression in CCA was related to the carcinoembryonic antigen (CEA) level but independent of the carbohydrate antigen (CA)19–9 level, Child‐Pugh grading, and vascular invasion (Figure 3B). In addition, results of IHC analysis available from the HPA database showed that HPCAL1 was more highly expressed in CCA tumor tissues than in the normal bile duct (Figure 3C). Furthermore, the 1‐year and 3‐year AUCs of HPCAL1 expression were 0.753 and 0.714, respectively, for predicting survival, which indicated that HPCAL1 has great prognostic value (Figure 3D).

### 3.4 HPCAL1 upregulated in CCA cancer tissues based on RT‐qPCR and western blot

**FIGURE 4:**
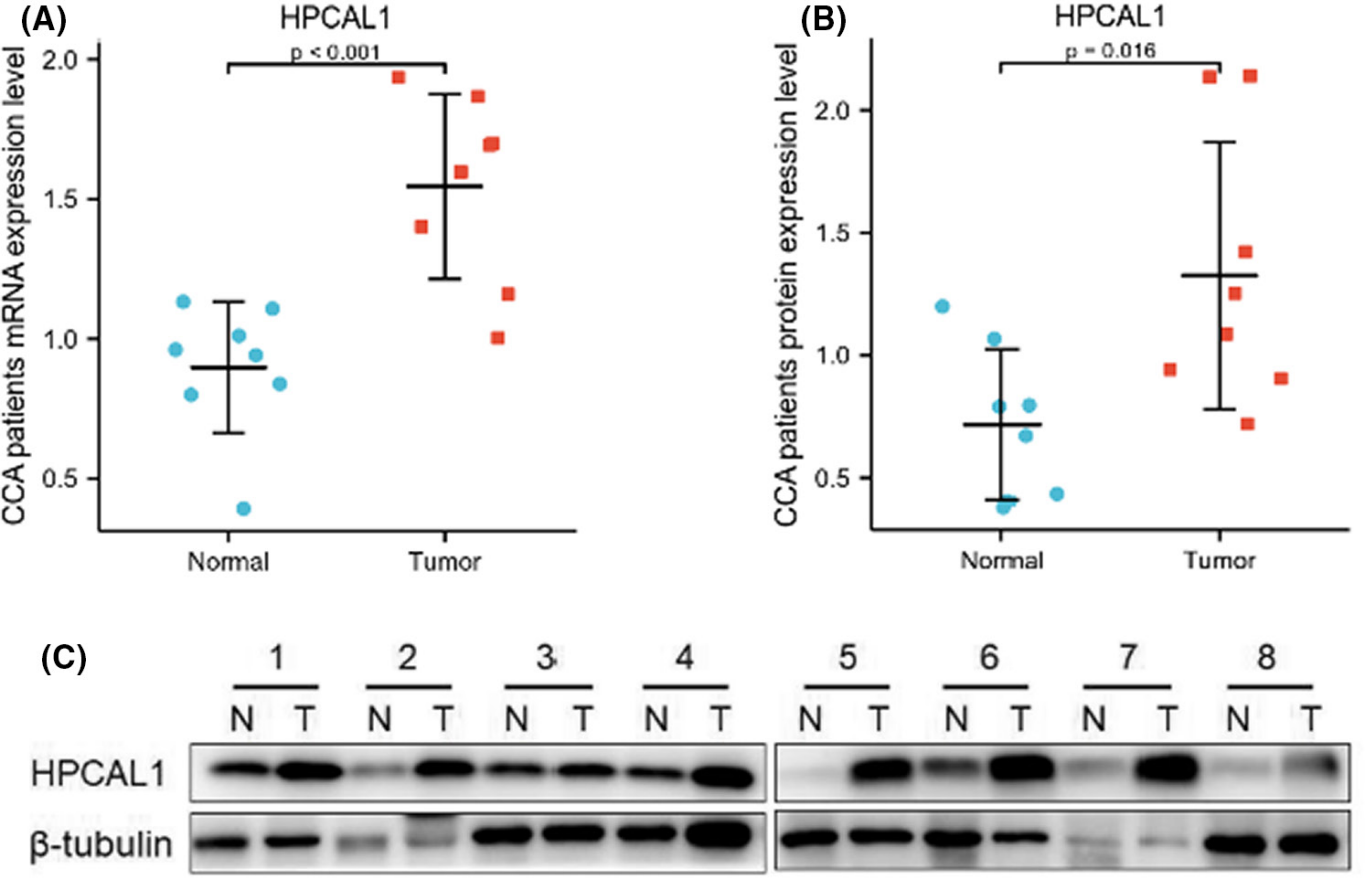
The expression of HPCAL1 in CCA patients. (A) mRNA expression of HPCAL1 in CCA patients by RT‐qPCR. (B, C) Protein expression of HPCAL1 in CCA patients by western blot.

RT‐qPCR was performed to validate the HPCAL1 mRNA levels within the eight pairs of CCA tissues. Results demonstrated that HPCAL1 was elevated in CCA compared with that in paracancerous tissues (marked as normal, Figure 4A). The protein expression of HPCAL1 was also dramatically and consistently increased as shown on western blot (Figure 4B,C).

### 3.5 IHC showing HPCAL1 as an independent prognostic marker of CCA

**FIGURE 5:**
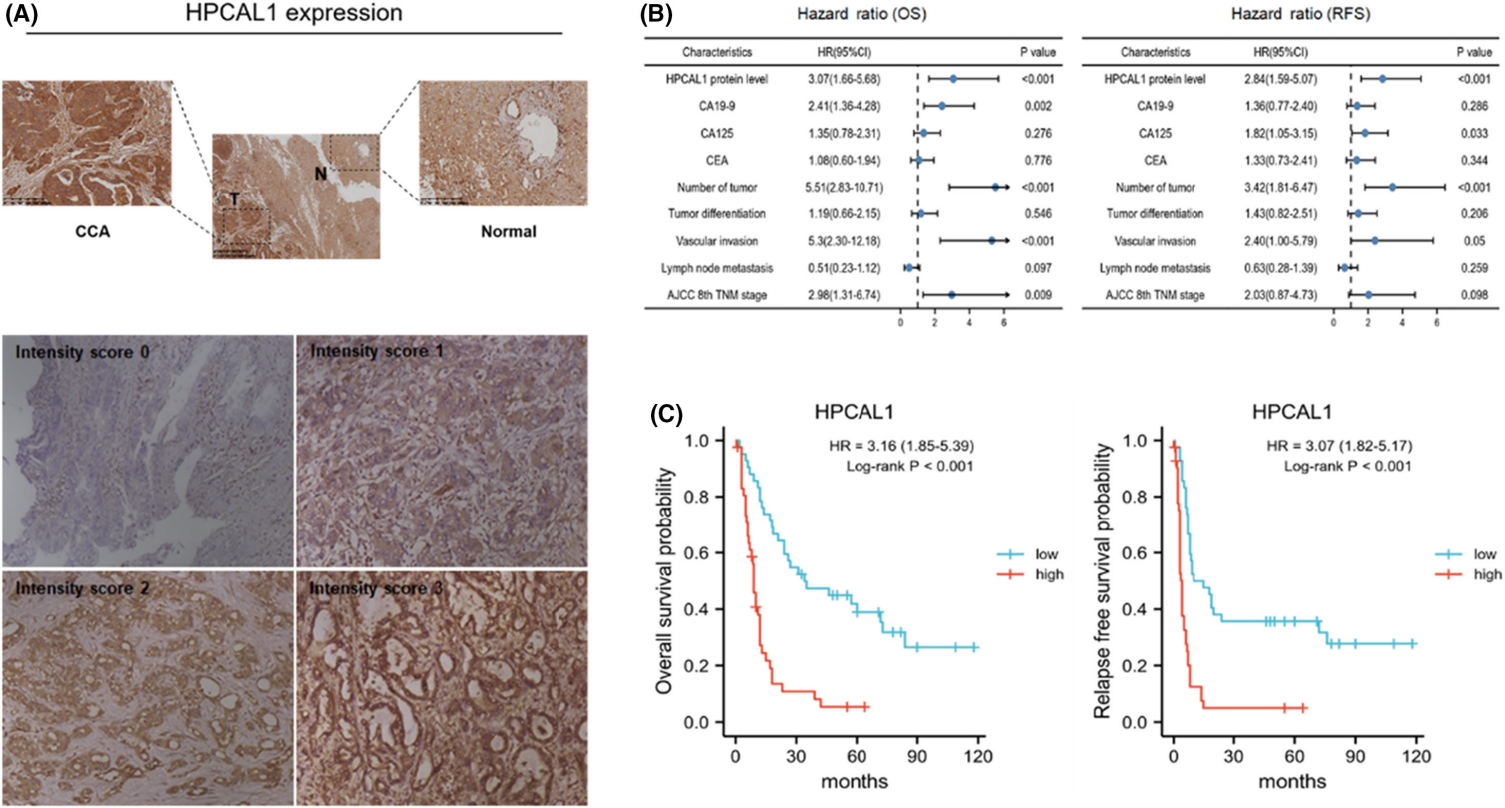
Prognostic gene signature of HPCAL1 in CCA patients. (A) Representative of IHC for HPCAL1, scoring from 0 to 3. (B) Multivariate Cox analysis of clinicopathological variables and OS/RFS. (C) Kaplan–Meier analysis of overall survival and relapse‐free survival in CCA patients with different levels of HPCAL1.

The clinical significance of HPCAL1 in 84 patients with CCA was evaluated and patients' characteristics are summarized in Table 1. Based on the results of IHC analysis, HPCAL1 was enriched in CCA tissues with positive staining, which was rarely expressed in normal tissues (Figure 5A upper). An obvious variation in the levels of HPCAL1 was detected in tumor samples (intensity scores from 0 for absent, 1 for weak, 2 for moderate, and 3 for strong) (Figure 5A lower). Univariate analysis indicated that nine variables correlated with the prognostic factors for OS and RFS. These variables were HPCAL1, CA19‐9, CA‐125, CEA, tumor number, tumor differentiation, vascular invasion, lymph node metastasis, and TNM stage. Furthermore, results of the multivariate analysis suggested that HPCAL1, CA‐125, tumor number, and tumor vascular invasion were prognostic factors for RFS, whereas HPCAL1, CA19‐9, number of tumors, tumor vascular invasion, and TNM stage were prognostic factors for OS (Table 2). The hazard ratios are summarized using a forest plot (Figure 5B). In terms of the correlation between HPCAL1 and clinicopathological features, the patients were divided into two groups: those with high HPCAL1 expression (HPCAL1 > 4.5, *n* = 42) and those with low HPCAL1expression (HPCAL1 ≤ 4.5, *n* = 42) (Table 3). This finding suggests that the higher the HPCAL1 expression in CCA patients, the greater the CA‐125 level (*p* = 0.04), number of tumors (*p* = 0.04), lymph node invasion (*p* = 0.02), and TNM stage (*p* = 0.04). The Kaplan–Meier analyses also indicated that patients with high HPCAL1 expression had shorter OS and higher recurrence rates than those with low expression (Figure 5C), which further highlights the crucial role of HPCAL1 in CCA progression.

**TABLE 1:** Description of ICC patients' data

Characteristics	Values
Age, year (mean ± SD)	57.00 ± 10.89
Gender (male/female)	45/39
ECOG score (≤1/>1)	75/9
ASA classification (I‐II/III)	69/15
Biliary tract stone (−/+)	61/23
Liver fluke (−/+)	80/4
HBsAg (−/+)	66/18
HBcAb (−/+)	32/52
CA19‐9 (≤35 U/L/>35 U/L)	33/51
CA125 (≤35 U/L > 35 U/L)	51/33
CEA (≤5 μg/L/>5μg/L)	50/34
Child‐Pugh (A/BC)	75/9
Tumor size (≤5 cm/>5 cm)	40/44
Number of tumor (single / multiple)	62/22
Tumor differentiation (<moderate/≥moderate)	28/56
Vascular invasion (−/+)	73/11
Bile duct invasion (−/+)	67/17
Nerve invasion (−/+)	76/8
Lymph node metastasis (−/+)	50/34
Adjacent organ invasion (−/+)	68/16
AJCC 8th TNM stage(I II/III IV)	39/45
Overall survival, months (median, 95% CI)	13.0 (10.5, 19.5)
Tumor recurrence, months (median, 95% CI)	6.5 (4.5, 8.0)

**TABLE 2:** Prognostic factor for RFS and OS of patients with ICC determined by using univariate and multivariate

Variable	Disease‐free survival			Overall survival		
	HR	95% CI	*p* value	HR	95% CI	*p* value
Univariate						
Age (≤57 or > 57)	0.99	0.96–1.01	0.36	0.99	0.96–1.01	0.45
Gender(male/female)	1.06	0.65–1.72	0.79	1.17	0.72–1.91	0.52
ECOG score (≤1/>1)	0.92	0.39–2.14	0.85	0.99	0.42–2.31	0.99
ASA classification (I‐II/III)	1.61	0.60–2.21	0.65	1.31	0.68–2.52	0.40
Biliary tract stone (−/+)	1.23	0.73–2.06	0.43	1.11	0.65–1.89	0.70
Liver fluke (−/+)	1.09	0.397–3.01	0.86	1.00	0.36–2.77	0.99
HBsAg (−/+)	1.06	0.60–1.89	0.82	1.01	0.56–1.83	0.96
HBcAb (−/+)	1.28	0.76–2.11	0.33	1.31	0.79–2.19	0.28
HPCAL1 protein level (≤4.5/>4.5)	3.48	2.07–5.83	**0.00**	3.78	2.21–6.48	**0.00**
CA19‐9 (≤35 U/L/>35 U/L)	2.12	1.25–3.59	**0.00**	2.66	1.54–4.58	**0.00**
CA125 (≤35 U/L > 35 U/L)	2.19	1.34–3.56	**0.00**	2.09	1.28–3.43	**0.00**
CEA (≤5 μg/L/>5 μg/L)	2.00	1.22–3.26	**0.00**	1.92	1.16–3.17	**0.01**
Child‐Pugh (A/BC)	1.78	0.81–3.92	0.14	2.01	0.95–4.24	0.06
Tumor size (≤5 cm/>5 cm)	1.24	0.77–2.02	0.36	1.21	0.74–1.98	0.43
Number of tumor (single/multiple)	3.67	2.08–6.48	**0.00**	3.86	2.21–6.72	**0.00**
Tumor differentiation (<moderate/≥moderate)	1.95	1.17–3.22	**0.01**	1.83	1.10–3.05	**0.02**
Vascular invasion (−/+)	3.12	1.48–6.58	**0.00**	4.49	2.20–9.13	**0.00**
Bile duct invasion (−/+)	1.21	0.65–2.22	0.53	1.60	0.88–2.91	0.12
Nerve invasion (−/+)	0.93	0.42–2.05	0.87	1.21	0.55–2.67	0.62
Lymph node metastasis (−/+)	1.93	1.18–3.16	**0.01**	2.04	1.24–3.38	**0.00**
Adjacent organ invasion (−/+)	1.53	0.84–2.76	0.15	1.40	0.74–2.63	0.29
AJCC 8th TNM stage(I II/III IV)	2.08	1.26–3.43	**0.00**	2.31	1.39–3.85	**0.00**
Multivariate						
HPCAL1 protein level (≤4.5/>4.5)	2.84	1.59–5.07	**0.00**	3.07	1.66–5.68	**0.00**
CA19‐9 (≤35 U/L/>35 U/L)	1.36	0.77–2.40	0.28	2.41	1.36–4.28	**0.00**
CA125 (≤35 U/L > 35 U/L)	1.82	1.05–3.15	**0.03**	1.35	0.78–2.31	0.27
CEA (≤5 μg/L/>5 μg/L)	1.33	0.73–2.41	0.34	1.08	0.60–1.94	0.77
Number of tumor (single/multiple)	3.42	1.81–6.47	**0.00**	5.51	2.83–10.71	**0.00**
Tumor differentiation (<moderate/≥moderate)	1.43	0.82–2.51	0.20	1.19	0.66–2.15	0.54
Vascular invasion (−/+)	2.40	1.00–5.79	**0.05**	5.30	2.30–12.18	**0.00**
Lymph node metastasis (−/+)	0.63	0.28–1.39	0.25	0.51	0.23–1.12	0.09
AJCC 8th TNM stage(I II/III IV)	2.03	0.87–4.73	0.09	2.98	1.31–6.74	**0.01**

Bold values indicate statistically significant.

**TABLE 3:** Correlation between HPCAL1 expression and intrahepatic cholangiocarcinoma in 84 ICC patients

Characteristics	Number of patients		*p*‐value
	Low HPCAL1 expression	High HPCAL1 expression	
Gender			0.82
Male	22	23	
Female	20	19	
Age			0.82
≤57	24	23	
>57	18	19	
ECOG score			0.48
≤1	39	36	
>1	3	6	
ASA classification			0.25
I‐II	37	32	
III	5	10	
Biliary tract stone			0.46
−	29	32	
+	13	10	
Liver fluke			0.11
−	38	42	
+	4	0	
HBsAg			0.28
−	31	35	
+	11	7	
HBcAb			>0.9999
−	16	16	
+	26	26	
CA19‐9			0.11
≤35 U/L	20	13	
>35 U/L	22	29	
CA125			**0.04**
≤35 U/L	30	21	
>35 U/L	12	21	
CEA			0.65
≤5 μg/L	26	24	
>5 μg/L	16	18	
Child‐Pugh			0.15
A	40	35	
B/C	2	7	
Tumor size			0.38
≤5 cm	22	18	
>5 cm	20	24	
Number of tumor			**0.04**
Single	35	27	
Multiple	7	15	
Tumor differentiation			0.16
<moderate	31	25	
≥moderate	11	17	
Vascular invasion			0.74
−	37	36	
+	5	6	
Bile duct invasion			0.78
−	34	33	
+	8	9	
Nerve invasion			>0.9999
−	38	38	
+	4	4	
Lymph node invasion			**0.02**
−	30	20	
+	12	22	
Adjacent organ invasion			0.09
−	37	31	
+	5	11	
AJCC 8th TNM stage			**0.04**
I/II	24	15	
III/IV	18	27	

Bold values indicate statistically significant.

### 3.6 GO analysis and KEGG analysis of HPCAL1 co‐expressed‐related genes

**FIGURE 6:**
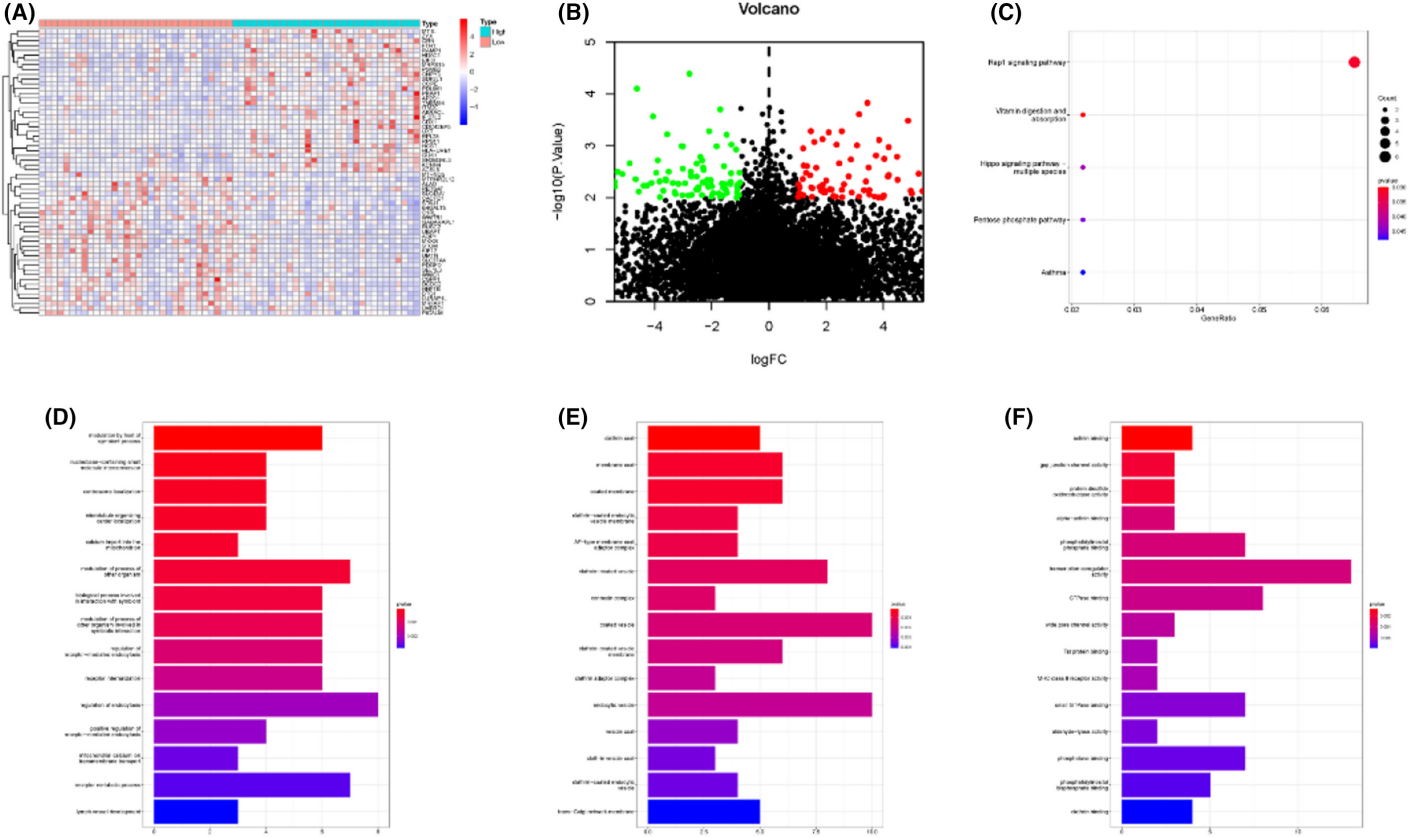
The GO and KEGG analysis of HPCAL1. (A) Heatmaps of the genes that are positively or negatively correlated with HPCAL1 (Top 30 genes are shown). (B) Volcanic map based on of co‐expressed‐related gens of HPCAL1. (C) KEGG pathway enrichment analyses of HPCAL1. (D) The GO term, biological processes of HPCAL1. (E) The GO term, cell components of HPCAL1. (F) The GO term, molecular functions of HPCAL1.

Lastly, the gene expression of the integrative microarray data was divided into two groups (high vs. low) based on the expression level of HPACL1, followed by HPCAL1 co‐expressed selection. The overall distribution of the co‐expressed‐related genes is displayed using a heatmap and volcano map (Figure 6A,B). Results of the GO analysis suggested that the expression of these genes was mainly concentrated in the modulation by a host of symbiont processes, nucleobase‐containing small molecule interconversion, centrosome localization, clathrin coat, membrane coat, coated membrane, actinin binding, gap junction channel activity, and protein disulfide oxidoreductase activity (Figure 6D–F). In addition, results of the KEGG analysis demonstrated that these genes were mainly involved in the Rap1 signaling pathway, vitamin digestion and absorption, and the Hippo signaling pathway (Figure 6C).

## 4 DISCUSSION

CCA, accounting for 15% of hepatobiliary malignancies, comprises a variety of malignancies in the intrahepatic or extrahepatic biliary tract.^1^ The incidence of CCA has increased worldwide in recent decades.^14^, ^15^ Despite the improvements in diagnosis and therapy, the recurrence rate after resection remains high, with a 5‐year survival of 7%–20%.^1^ Therefore, it is imperative to investigate the mechanisms underlying the development of CCA and to identify novel potential therapeutic targets. Advances in bioinformatics have been widely used to explore the genes associated with cancer development and progression. Da et al. proposed that CASK may be a tumor suppressor and an independent prognostic marker for CCA using bioinformatics combined with quantitative proteomics,^16^ while Zhang et al. identified 191 novel methylation‐regulated DEGs for CCA that were enriched in the cell cycle, nuclear division, xenobiotic metabolism, drug catabolism, and negative regulation of proteolysis based on the results of an integrated bioinformatics analysis.^17^

In this study, 13 overlapping DEGs were identified from two microarray datasets (GSE34166 and GSE22633). After examining the integrated TCGA‐CHOL/GEO‐GSE107943 database, all target expression tendencies were consistent, except for the expression of CDH6, KCNS1, MMP1, and ZNF503. Subsequently, the prognostic significance of these targets was evaluated. The high expression of CDH6 prolonged the OS; this finding was consistent with the results of a previous study, which showed that CDH6 was a potential tumor suppressor and target of epigenetically dysregulated miR‐429 in CCA,^18^ but was insignificantly correlated with prolonged RFS. Notably, a higher HPCAL1 expression was associated with shorter OS and RFS, indicating that it is an independent prognostic biomarker for CCA. Furthermore, the expression and prognostic value of HPCAL1 in CCA were confirmed in the validation cohort. High HPCAL1 expression in CCA patients is likely to indicate a more advanced TNM stage, higher CA‐125 levels, more tumors, and lymph node invasion. Additionally, the GO term and KEGG pathway analyses revealed that upregulated HPCAL1 was to be primarily enriched in the modulation by a host of symbiont processes, clathrin coat, actinin binding, and Rap1 signaling pathway.

In the past decade, many calcium‐sensing proteins have been identified in the CNS. These proteins were regrouped and named neuronal Ca2+ sensor (NCS) proteins. Previous studies have well documented the whole family of NCS proteins, and 14 NCS protein genes are known to exist in various species. Here, we mainly focused on the subfamily of visinin‐like proteins (VSNLs). The VSNLs were subdivided into five subfamilies. These include visinin‐like protein 1 (VILIP‐1) (VSNL1), VILIP‐2 (HPCAL4), VILIP‐3 (HPCAL1), hippocalcin (HPCA), and neurocalcin δ (NCALD).^10^ Visinin‐like protein 1 (VILIP‐1) was recently identified as a putative tumor migration suppressor gene.^19^, ^20^, ^21^, ^22^ A weaker or lower VILIP‐1 expression indicate more invasive features, such as the depth of tumor invasion and local lymph node metastasis in esophageal cancer, and poorer survival rate of aggressive non‐small cell lung carcinoma cell lines and primary tumors. Additionally, VILIP2, hippocalcin, and neurocalcin δ have rarely been reported in oncologic studies.

HPCAL1, a member of the VILIP superfamily, is a neuronal calcium sensor with three EF‐hand structures.^23^, ^24^, ^25^ HPCAL1 was associated with Alzheimer's disease,^10^ autism,^26^ hypertension,^27^ coronary heart disease,^28^ chronic kidney disease,^29^ and enteropathogenic *Escherichia coli* infection.^30^ Moreover, studies on HPCAL1 have recently attracted increasing attention in cancer research. HPCAL1 inhibits hepatocellular carcinoma progression by promoting p21 stabilization through the activation of the ERK1/2‐MAPK signaling pathway.^11^ The miR204‐HPCAL1‐LncRNAHOTTIP pathway is probably involved in the carcinogenic progression of HCV core‐related HCC disease.^31^ By contrast, HPCAL1 binding contributes to the differentiation and neurite outgrowth of neuroblastoma cells, and aberrant HPCAL1 expression may lead to cancer malignancy by impeding the differentiation of immature sympathetic neurons.^12^ HPCAL1 also activates the Wnt/β‐catenin pathway by enhancing ERK stimulation and inhibiting GSK3β, thereby contributing to the proliferation of glioblastoma.^32^ Our results, partly in line with the findings of the abovementioned studies, showed that HPCAL1 functions as a tumor‐promoting oncogene in CCA. Additionally, HPCAL1 is a serum marker for pancreatic cancer diagnosis,^33^, ^34^ methylation changes in CNT‐induced lung cancer,^35^ and a highly plastic epigenetic marker of the early origin of prostate cancer.^36^ Consistently, our findings showed that the high expression of HPCAL1 was associated with worse OS and RFS in CCA and may be a promising biomarker of CCA prognosis.

Moreover, the functional enrichment analyses of HPCAL1 co‐expressed‐related genes indicated that HPCAL1 may be involved in actinin binding and the Rap1 signaling pathway. Actinin binding plays a vital role in cancer development. Moreover, actinin‐4 expression is significantly increased during the development of cancer, such as colorectal and pancreatic cancers. Actinin‐4 not only promotes tumor cell growth, but also drives tumor cell invasion and metastasis.^37^, ^38^ Rap1 is a potentially significant modulator of oncogenic pathways in various tumors.^39^, ^40^ Overall, HPCAL1 may be an essential biomarker that affects the occurrence and development of CCA.

Through the bioinformatics analysis, this study not only identified a tumor suppressor gene of CCA, CDH6, which has been confirmed by relevant basic studies, but also found a tumor‐promoting oncogene, HPCAL1, which was associated with poor prognosis of CCA and was confirmed by our validation cohort. However, this study has some limitations. First, most data were obtained from a publicly available database and validated in our single‐center cohort. Hence, controlled and multicenter clinical trials are warranted to more accurately understand the role of HPCAL1 in CCA. Second, the relationship between HPCAL1 and efficacy of radio‐chemotherapy should be further explored. Third, in vivo and in vitro validation and further molecular experiments are required to explore the underlying mechanisms of HPCAL1 in CCA.

To the best of our knowledge, this study was the first to identify HPCAL1 as a new biomarker for CCA. HPCAL1 is upregulated in CCA tissues. The increased expression of HPCAL1 in CCA patients potentially indicates poor prognosis. Further research on the functional impact of HPCAL1 using biomolecular research methodologies would be helpful in providing more evidence of the prognostic target application of HPCAL1.

## AUTHOR CONTRIBUTIONS

Conceptualization, Mingjian Ma and Guangyan Zeng; Formal analysis, Jinhui Li; Data curation, Jiahua Liang; Funding acquisition, Jiancong Chen; Software, Jiahua Liang; Supervision, Li Huang and Jiaming Lai; Writing‐original draft, Mingjian Ma and Guangyan Zeng; Writing‐review & editing, Jinhui Li and Jiancong Chen. All authors read the final manuscript and provide their consent for publication.

## CONFLICT OF INTEREST

The authors declare that they have no known competing financial interests or personal relationships that could have appeared to influence the work reported in this paper.

## ETHICAL STATEMENT

The study was also in accordance with the Helsinki Declaration and approved by the Ethics Committee of the First Affiliated Hospital of Sun Yat‐sen University [No. (2021)170]. Written informed consents were obtained from all patients.

## Supporting information


Figure S1‐S3

## Data Availability

Data sharing is not applicable to this article as no new data were created or analyzed in this study.
