# Supplementary material for: Expressional and prognostic value of HPCAL1 in cholangiocarcinoma via integrated bioinformatics analyses and experiments
Source: Cancer Med. 2022 May 29;12(1):824–36. doi: 10.1002/cam4.4897 (PMC9844623; doi:10.1002/cam4.4897)
Supplement: Supplementary file 1 — Figure S1‐S3 [file CAM4-12-824-s001.docx]

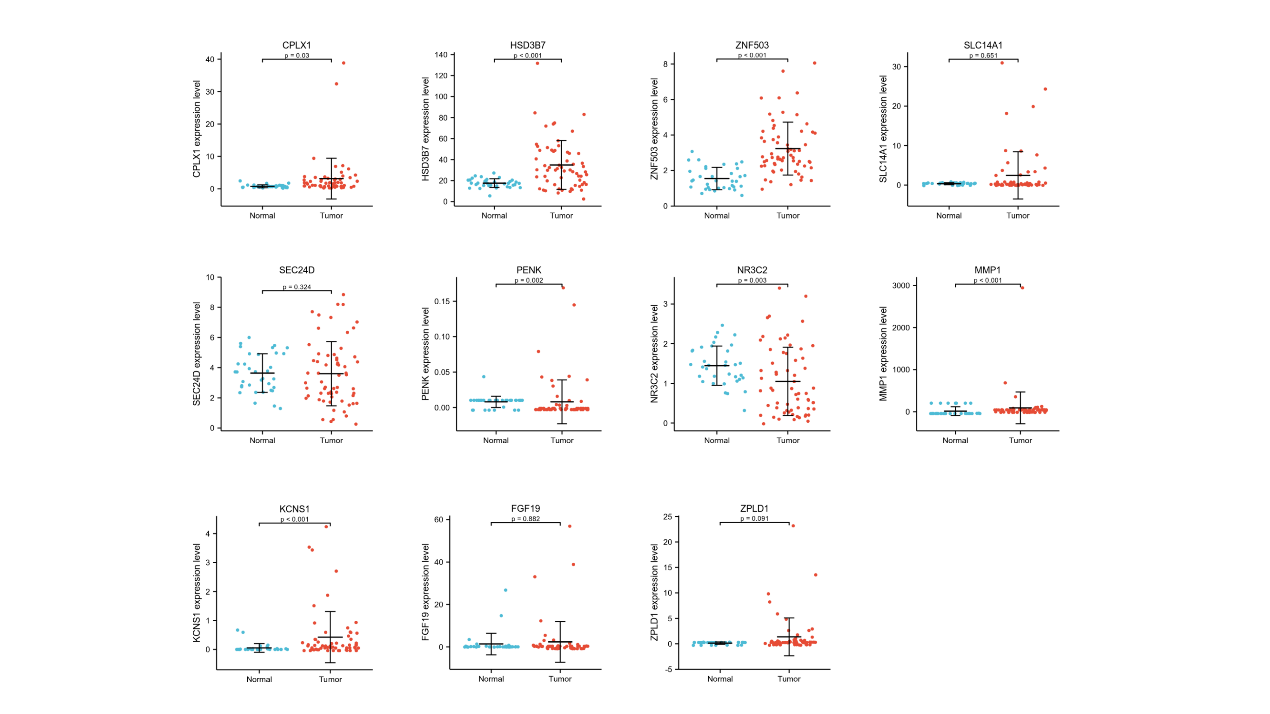


**Supplementary Figure 1:** Comparison of CPLX1, HSD3B7, ZNF503, SLC14A1, SEC24D, PENK, NR3C2, MMP1, KCNS1 and FGF19 levels in normal and tumor tissues.


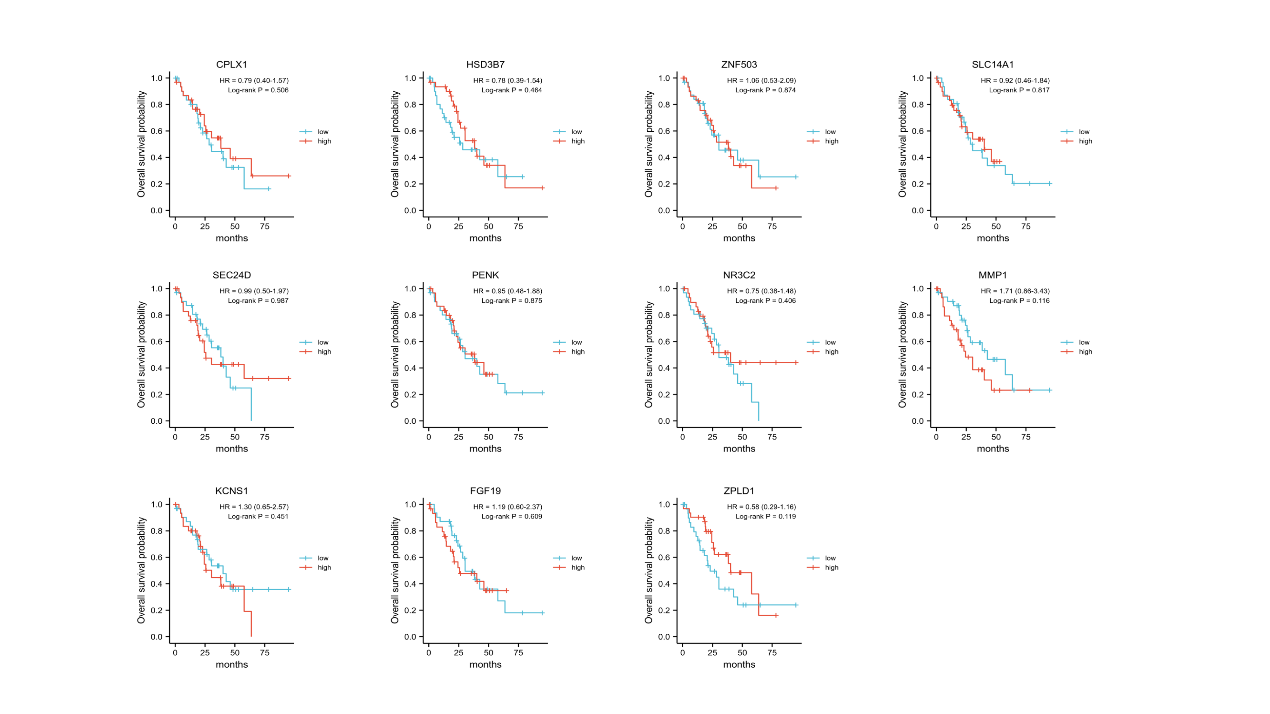


**Supplementary Figure 2:** The overall survival analysis of CPLX1, HSD3B7, ZNF503, SLC14A1, SEC24D, PENK, NR3C2, MMP1, KCNS1 and FGF19 expression in integrated TCGA/GEO database.


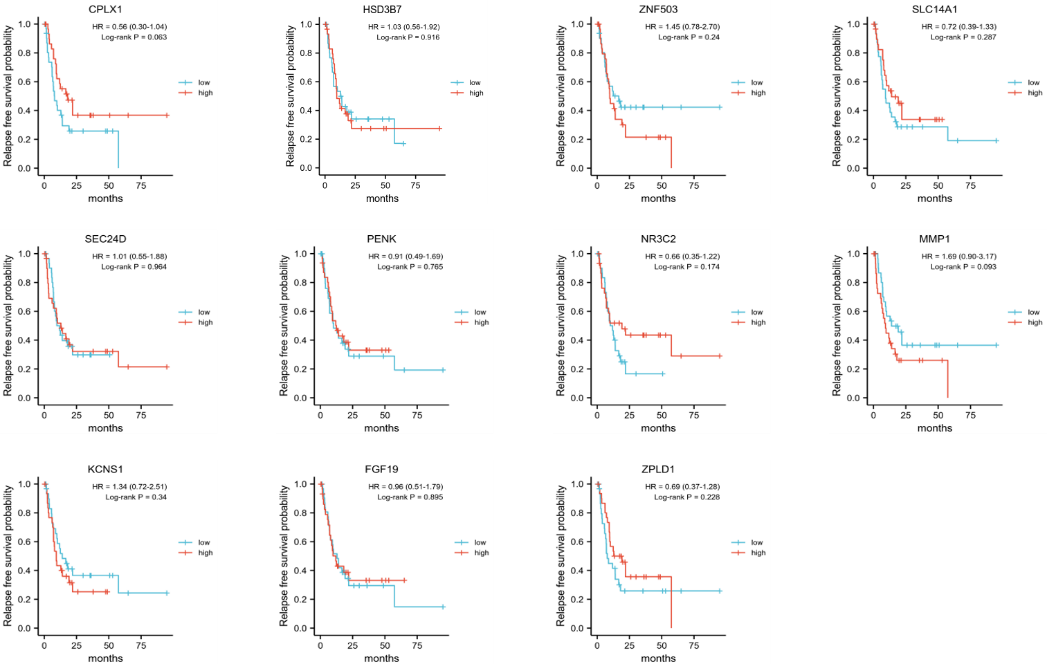


**Supplementary Figure 3:** The relapse free survival analysis of CPLX1, HSD3B7, ZNF503, SLC14A1, SEC24D, PENK, NR3C2, MMP1, KCNS1 and FGF19 expression in integrated TCGA/GEO database.
